# Examining the Efficacy of Extended Reality–Enhanced Behavioral Activation for Adults With Major Depressive Disorder: Randomized Controlled Trial

**DOI:** 10.2196/52326

**Published:** 2024-04-15

**Authors:** Margot Paul, Kim Bullock, Jeremy Bailenson, David Burns

**Affiliations:** 1 Department of Psychiatry and Behavioral Sciences Stanford University School of Medicine Stanford, CA United States; 2 Department of Communication Stanford University Stanford, CA United States

**Keywords:** virtual reality, extended reality, major depressive disorder, behavioral activation, depression, Meta Quest 2

## Abstract

**Background:**

Major depressive disorder (MDD) is a global concern with increasing prevalence. While many evidence-based psychotherapies (EBPs) have been identified to treat MDD, there are numerous barriers to patients accessing them. Virtual reality (VR) has been used as a treatment enhancement for a variety of mental health disorders, but few studies have examined its clinical use in treating MDD. Behavioral activation (BA) is a simple yet effective and established first-line EBP for MDD that has the potential to be easily enhanced and adapted with VR technology. A previous report by our group explored the feasibility and acceptability of VR-enhanced BA in a small clinical proof-of-concept pilot. This study examines the clinical efficacy of a more immersive extended reality (XR)–enhanced BA (XR-BA) prototype. This is the first clinical efficacy test of an XR-BA protocol.

**Objective:**

This study examined whether XR-BA was feasible and efficacious in treating MDD in an ambulatory telemedicine clinic.

**Methods:**

A nonblinded between-subject randomized controlled trial compared XR-BA to traditional BA delivered via telehealth. The study used a previously established, brief 3-week, 4-session BA EBP intervention. The experimental XR-BA participants were directed to use a Meta Quest 2 (Reality Labs) VR headset to engage in simulated pleasant or mastery activities and were compared to a control arm, which used only real-life mastery or pleasant activities as between-session homework. The Patient Health Questionnaire (PHQ)–9 was the primary outcome measure. Independent-sample and paired-sample *t* tests (2-tailed) were used to determine statistical significance and confirmed using structural equation modeling.

**Results:**

Overall, 26 participants with MDD were randomized to receive either XR-BA (n=13, 50%) or traditional BA (n=13, 50%). The mean age of the 26 participants (n=6, 23% male; n=19, 73% female; n=1, 4% nonbinary or third gender) was 50.3 (SD 17.3) years. No adverse events were reported in either group, and no substantial differences in dropout rates or homework completion were observed. XR-BA was found to be statistically noninferior to traditional BA (t_18.6_=−0.28; *P*=.78). Both the XR-BA (*t*_9_=2.5; *P*=.04) and traditional BA (t_10_=2.3; *P*=.04) arms showed a statistically significant decrease in PHQ-9 and clinical severity from the beginning of session 1 to the beginning of session 4. There was a significant decrease in PHQ-8 to PHQ-9 scores between the phone intake and the beginning of session 1 for the XR-BA group (t_11_=2.6; *P*=.03) but not the traditional BA group (t_11_=1.4; *P*=.20).

**Conclusions:**

This study confirmed previous findings that XR-BA may be a feasible, non-inferior, and acceptable enhancement to traditional BA. Additionally, there was evidence that supports the potential of XR to enhance expectation or placebo effects. Further research is needed to examine the potential of XR to improve access, outcomes, and barriers to MDD care.

**Trial Registration:**

ClinicalTrials.gov NCT05525390; https://clinicaltrials.gov/study/NCT05525390

## Introduction

### Background

Major depressive disorder (MDD) is a global concern with increasing cases worldwide [1]. Depressive disorders are the most significant contributors to nonfatal health loss worldwide, with a 37.9% increase in their economic burden from 2010 to 2020 [1,2]. MDD is associated with suicide, which is one of the leading causes of death in young adults. Although many evidence-based psychotherapies (EBPs) for MDD exist, less than 1 in 4 people in low- to middle-income countries receive these treatments [1]. Thus, creating solutions for access to care is a priority in the treatment of MDD.

Due to its simplicity and efficacy, behavioral activation (BA) is one of the most widely used first-line EBP for MDD [3]. The behavioral theory underpinning this intervention postulates that depression is due to an avoidance pattern of increasingly less frequent engagement in pleasurable or mastery activities [3,4]. BA provides the tools for reversing this pattern through intentional scheduling of and engaging in positive and mastery activities. Despite BA’s usefulness and effectiveness, few patients with MDD ever obtain access to BA due to barriers and obstacles such as physical limitations, financial constraints to accessing activities, social isolation, mental health stigma, or a lack of trained providers [5,6].

Extended reality (XR) is a term that is used to describe all current and future immersive technologies, including virtual reality (VR) and augmented reality. XR is becoming increasingly popular, with approximately 1 in 5 consumers in the United States using it in 2020 and an estimated 70.8 million people in the United States using it at least once per month in 2023 [7]. Immersive technologies such as VR are being used to solve multiple barriers to mental health care, such as improving access to content not readily accessible in real life (IRL). The use of VR to enhance the treatment of anxiety and trauma disorders has been reported on for the past 3 decades owing to its ability to easily and reliably provide controlled cue desensitization [8].

While there is a preponderance of support illustrating XR’s efficacy in enhancing EBP and reducing barriers to care, surprisingly few clinical studies have examined its use directly in populations with MDD [8,9]. While several studies have examined the use of VR to treat mood disorders, to our knowledge, only 1 clinical trial focusing on using VR-enhanced BA (VR-BA) in a population with MDD has been completed to date [9,10]. This previous feasibility study, completed by our group, observed evidence of improvement in MDD outcomes using VR to simulate pleasant activities during a brief BA intervention [10].

This study is an extension of our previous VR-BA feasibility study and remains in line with the international working group’s methodological framework for developing the design, implementation, analysis, interpretation, and communication of trials of novel VR behavioral health treatments [11]. We iterated our previous VR-BA prototype based on pilot-testing and feedback to a more immersive, embodied, and autonomous XR prototype (XR-enhanced BA [XR-BA]) using a commercially available Meta Quest 2 (Reality Labs) VR headset. On the basis of user feedback, we made the XR-BA protocol identical to traditional in vivo or IRL BA by allowing patients to freely decide which XR pleasant activities to engage in rather than being restricted to a list of curated pleasant experiences. This allowed for a more personalized customization and a higher sense of autonomy by users and mirrors the elements associated with traditional BA. It also more closely mimics traditional BA by refraining from confining participants to preselected VR choices. While the devices were not Health Insurance Portability and Accountability Act–compliant at this time, participants were informed of the potential privacy risks associated with Meta potentially tracking their use. However, the authors and the institutional review board considered this risk akin to that of traditional BA, where activities can be observed by others or tracked through purchases. Furthermore, no mental health–specific data were collected on the device.

This study went beyond examining simple feasibility and tested the clinical efficacy of XR-BA compared to traditional BA. We specifically examined whether XR-BA is efficacious in reducing clinical depressive symptoms and shows noninferiority to traditional BA delivered without VR in an ambulatory MDD sample.

### Objectives

The first aim of this study was to test the safety and feasibility of our newest prototype (XR-BA) using an embodied and interactive VR headset to engage with maximum free choice during a brief BA protocol guided by a telehealth clinician. It was hypothesized that XR-BA would be safe, feasible, and acceptable for outpatients with MDD receiving remote care.

The second aim of this study was to examine the efficacy of XR-BA compared to that of a traditional brief BA protocol for MDD. We predicted from prior work that XR-BA would not be inferior to traditional brief BA in reducing symptoms of MDD as measured using the Patient Health Questionnaire–9 (PHQ-9). The decision to compare XR-BA to a traditional BA protocol rather than a sham VR control was because engagement with VR itself can be considered a pleasant activity within BA, and thus could be a confounding variable within a sham control. Thus, with traditional BA being the gold EBP standard with known efficacy, it seemed a more meaningful comparison. If XR-BA can be as efficacious as traditional BA and more accessible to those with barriers to IRL pleasant activities, then it may prove to be an impactful enhancement to treatment.

## Methods

### Recruitment

Recruitment took place remotely via Zoom (Zoom Video Communications)–delivered telehealth sessions between December 19, 2022, and July 24, 2023. The trial was registered on ClinicalTrials.gov (ID NCT05525390).

Participants were recruited locally via study flyers posted in the Stanford School of Medicine Department of Psychiatry and Behavioral Sciences located in Palo Alto, California, United States. The description of the study was also electronically listed on Stanford University’s website for currently recruiting studies, ClinicalTrials.gov, and Craigslist. Without solicitation, a private web-based company called *Power* included our study on its website and connected participants with this study without any formal agreement, consent, or payment from our research group.

The inclusion criteria were as follows: age of ≥18 years; ability to speak English; and meeting of the *Diagnostic and Statistical Manual of Mental Disorders, Fifth Edition*, criteria for MDD. The exclusion criteria were as follows: substance use disorder in the previous year, diagnosis of any psychotic or bipolar I disorder, seizure in the previous 6 months or untreated epilepsy, current suicidal urges or intent, current nonsuicidal self-injury or parasuicidal behavior, changing psychotherapy treatment within the last 4 months before study entry, or changing psychotropic medication within 2 months of study entry. This study offered no compensation for participation.

The initial screening procedure consisted of 2 steps: an initial phone screening and a face-to-face Zoom intake session. During the initial phone screening, callers were assessed for preliminary eligibility using the Patient Health Questionnaire–8 (PHQ-8) and a brief screening questionnaire and were given the opportunity to ask questions about the study (Multimedia Appendix 1). If the initial eligibility criteria were met, as determined by the answers to the questionnaire and a PHQ-8 score of ≥10, potential participants were securely emailed a consent form to read, review, and sign at their leisure [12] (Multimedia Appendix 2). Potential participants were informed that they could reach out to the clinician with any questions before signing the consent form. After potential participants securely returned their signed consent forms, a Zoom intake session was held to determine complete study eligibility and obtain demographic information (Multimedia Appendix 3). Complete study eligibility was determined using the clinician-administered Mini-International Neuropsychiatric Interview [13]. The previously published case report and feasibility study provide further details [10,14].

### Enrollment and Randomization

When a participant met the full study eligibility criteria and was enrolled to take part in the study, they were randomly assigned to 1 of the 2 study arms in a single-blind fashion using permuted blocks of 4 in sealed envelopes. Participants were notified of their randomization outcome via secure email before session 1.

#### Procedure

A clinical psychologist met with each participant for 30 to 50 minutes once per week for 4 sessions over Zoom to administer a brief BA therapy protocol. At the beginning of each session, all participants were verbally administered the PHQ-9. If item 9 was endorsed, a risk assessment was conducted in real time, and safety measures were taken in accordance with the risk. Both arms followed the protocol for brief BA based on the guidance of the published literature [15,16]. No participants were provided with a stipend for activities. All sessions followed the previously established protocol detailed in the case report and feasibility study [10,14].

#### Experimental Arm (XR-BA)

The XR-BA participants were shipped a VR Meta Quest 2 headset before the first session with a prepaid return label. This headset has a resolution of 1832 × 1920 pixels; support for a 60-, 72-, and 90-Hz refresh rate; and room scale [17].

The Meta Quest 2 headsets did not have any software preloaded or prechosen. XR-BA participants were provided with an XR activity list similar to the Pleasant Events Schedule from traditional BA. The items included in the list were determined by asking subject matter experts to provide quality activity choices based on the categories provided within the Meta Quest 2 headset. While the list included different category options and ideas from those within the Meta Quest 2 headset, the clinician clarified that participants could choose any activity offered within the headset even if it was not included in the list (Multimedia Appendix 4) [18]. In between each session, participants were asked to complete ≥4 XR activities per week and 1 post-XR questionnaire pertaining to all completed XR activities from the week to assess spatial presence, simulator sickness (tolerability), and technology acceptability (Multimedia Appendix 5). This questionnaire was sent out to the participant and returned to the clinician via a secure email.

#### Control Arm (Traditional BA)

The participants in the control group followed the same protocol as those in the XR-BA arm except that they were not provided with a VR headset, were emailed the Pleasant Events Schedule, were asked to choose and complete ≥4 activities IRL, and were not administered the post-XR questionnaire.

**Figure 1:**
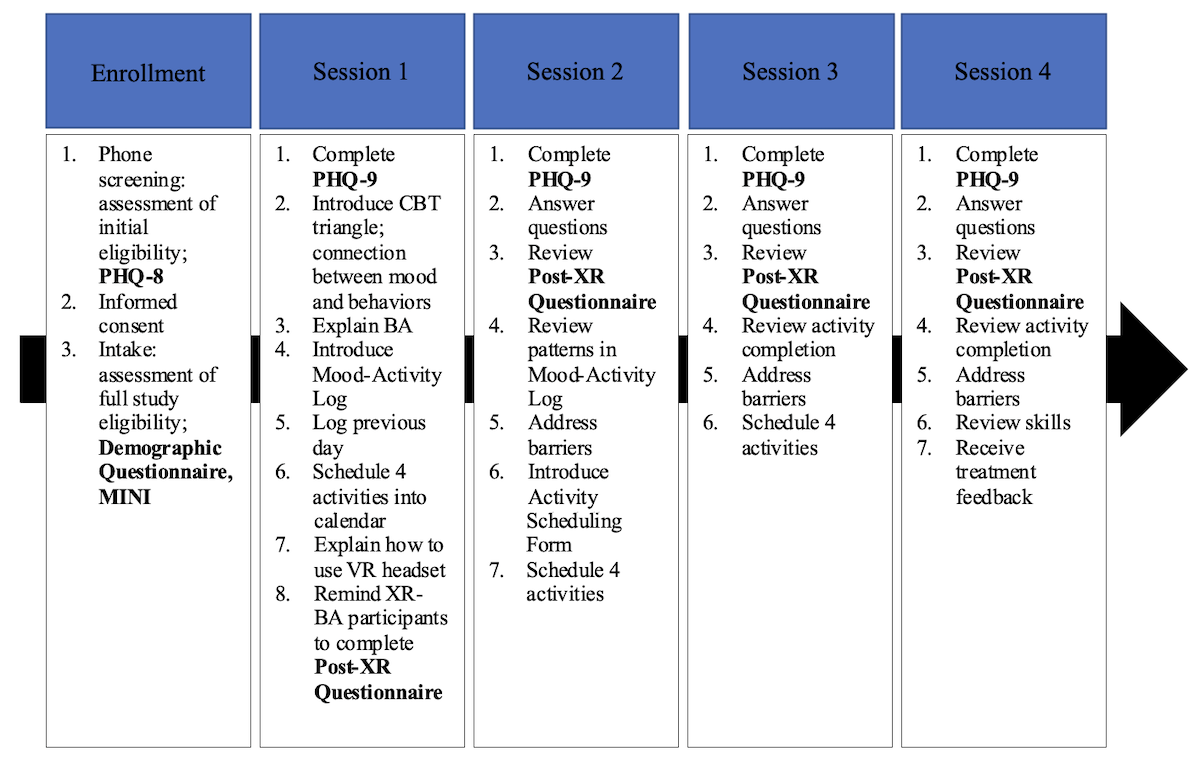
Study timeline. BA: behavioral activation; CBT: cognitive behavioral therapy; MINI: Mini-International Neuropsychiatric Interview; PHQ-8: Patient Health Questionnaire–8; PHQ-9: Patient Health Questionnaire–9; VR: virtual reality; XR: extended reality; XR-BA: extended reality–enhanced behavioral activation.

The previously published case report and feasibility study provide more details [10,14]. The study timeline is shown in Figure 1.

### Measures

The feasibility, or the degree to which XR could successfully be integrated into the brief BA protocol, was measured by commenting on qualitative barriers to use observed. Barriers were assessed by rates of dropout, adverse events, number of times the headset was used, and level of presence experienced in the headset [11]. The level of presence was obtained via participant reports using a Likert scale of 0 (*not at all*) to 4 (*very strongly*) for each question; with 3 questions, there was a possibility of yielding a score between 0 and 12.

The acceptability of the XR-BA treatment was measured via participant reports using the Technology Acceptance Model, with the agreement choices on a Likert scale ranging from 0 (*strongly disagree*) to 4 (*strongly agree*) for each question; with 3 or 4 questions per category, there was a possibility of yielding a score between 0 and 12 or 0 and 16, respectively.

The tolerability of the XR-BA treatment was measured via participant reports using the Simulator Sickness Questionnaire (SSQ), with the agreement choices on a Likert scale ranging from 0 (*no more than usual*) to 3 (*severely more than usual*); with 16 items, there was a possibility of yielding a score between 0 and 48.

The efficacy of the XR-BA treatment was measured via participant reports using the PHQ-8 and PHQ-9, with the agreement choices on a Likert scale ranging from 0 (*not at all*) to 3 (*nearly every day*); with 8 or 9 questions, there was a possibility of yielding a score between 0 and 24 or 0 and 27, respectively.

The previously published case report [14] provides a more in-depth description and background of the following measures: demographic questionnaire, Mini-International Neuropsychiatric Interview, PHQ-8*,* PHQ-9, presence scale, Technology Acceptance Model, and SSQ.

Of note, agitation (ie, the brief agitation measure) was not used as a measure of tolerability in this study. In addition, unlike the previous study, the number of times the headset was used was not determined from the device itself; rather, it was obtained via participant self-report.

### Statistical Analyses

#### Overview

This study was a 2-arm nonblinded between-participant randomized controlled trial (RCT) testing the feasibility and efficacy of using XR-simulated activities compared to IRL pleasurable or mastery activities during a brief BA intervention for MDD. The Holter critical number in structural equation modeling (SEM) with the analysis of moment structures (AMOS; version 28.0; IBM Corp) [19] was used to determine whether a sample size of 26 would be needed to disprove the model if it were incorrect.

#### Feasibility

The average total presence for intention-to-treat (ITT) participants and protocol completers was calculated. The average presence experienced was also calculated as a percentage by dividing the average score by 12 (the maximum score). The number of questions in each category determined the outcome range (either 0-12 for 3 questions or 0-16 for 4 questions). The average percentage of acceptance was also calculated by dividing the average score by the maximum score within the outcome range. To determine the degree of acceptance, as labeled on the scale, the average score was then scaled back depending on the number of questions. For example, the “Perceived Usefulness” category included 3 questions, yielding a potential range of 0 to 12, so an average score of 10 would be divided by 3 to assess the degree of acceptance (in this case, it would yield a score of 3.33, which would correlate to “agree” on the Likert scale). Physical tolerability of the VR headset was assessed via participant reports using the SSQ, which was broken down by symptom and used a Likert scale ranging from 0 (*no more than usual*) to 3 (*severely more than usual*) for each item. The percentage of physical intolerability was calculated by dividing the average scores by the highest potential score (48).

#### Efficacy

To assess the clinical efficacy of the XR-BA treatment compared with the traditional BA treatment group, the participants’ depression scores were measured using the PHQ-8 from the initial phone screening and the PHQ-9 from the 4 session time points. Independent-sample *t* tests (2-tailed) were used to compare the means between the 2 groups, and paired-sample *t* tests (2-tailed) were used to compare the means within each group. In addition, SEM AMOS was used to confirm the results because of its ability to compare competing models using nested tests, compare parameter estimates across groups, and estimate missing data models using full-information maximum likelihood [19-21]. SEM is widely used in the social sciences and was chosen for this study given its ability to adeptly manage missing data and exhibit greater statistical power compared to conventional multiple regression analyses [22], which was important given this study’s relatively low sample size. The chi-square statistic was used to evaluate model fit [23]. Changes in chi-square values relative to changes in *df* (chi-square difference tests) were used to compare nested models. These results were also confirmed using traditional linear growth models [24].

### Ethical Considerations

Ethics approval was obtained from the Stanford University institutional review board (protocol 66488) and participants provided informed consent before beginning the study. Participant data were deidentified and participants were informed of the potential privacy risks associated with Meta potentially tracking their use. No mental health–specific data were collected on the device. Participants were not compensated for participation.

## Results

### Participant Demographics

The sample consisted of 26 adults (mean age 50.3, SD 17.3 y; n=6, 23% male; n=19, 73% female; and n=1, 4% nonbinary or third gender), with 21 (81%; mean age 47.9, SD 17.7 y; n=5, 24% male; n=15, 71% female; and n=1, 5% nonbinary or third gender) completing the full protocol. The Holter critical number in SEM AMOS was used to determine that a sample size of 26 would be needed to disprove the model if it were incorrect. There was no significant difference in age (t_23.6_=1.34; *P*=.19) or sex (t_23.4_=0.71; *P*=.49) between the groups.

**Figure 2:**
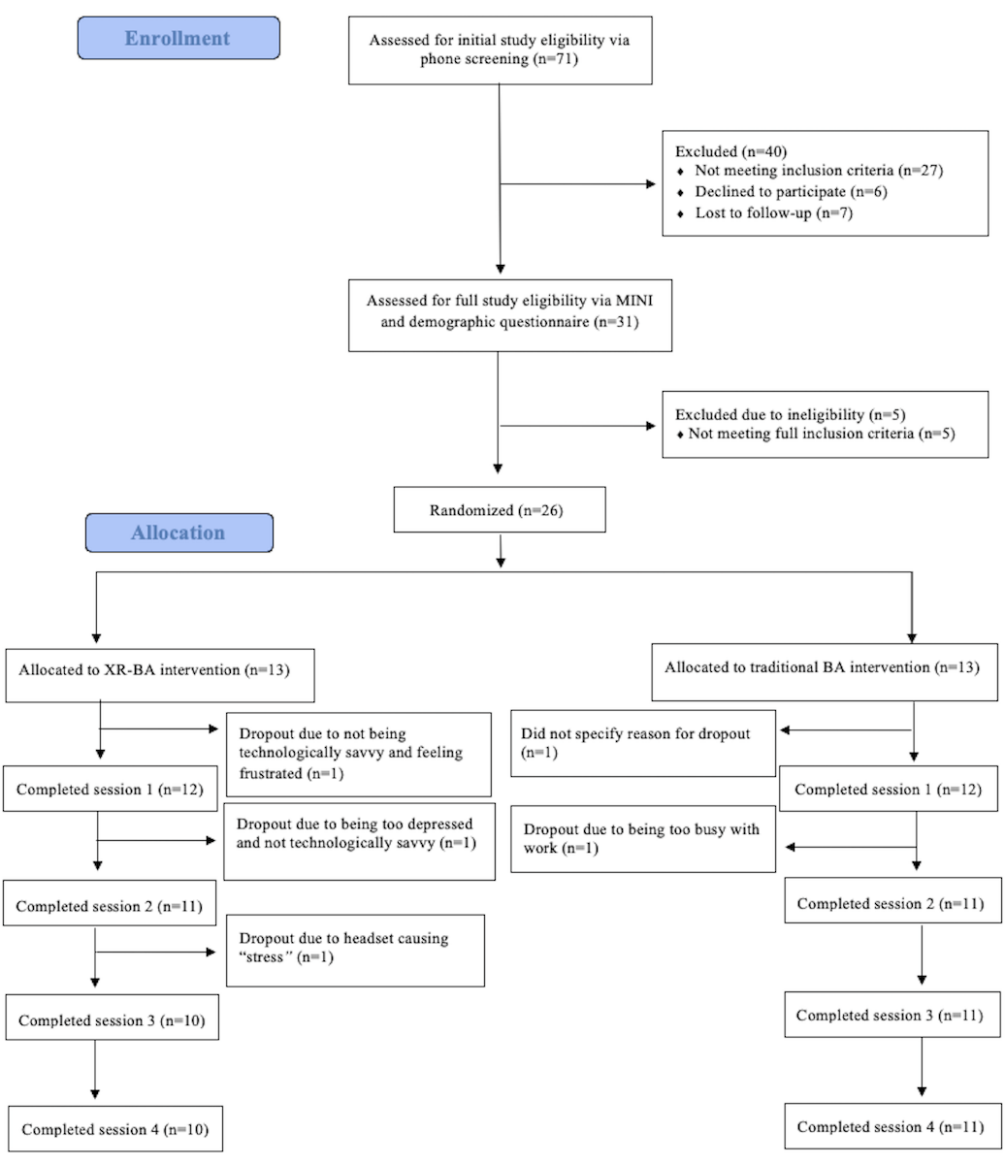
CONSORT (Consolidated Standards of Reporting Trials) diagram. BA: behavioral activation; MINI: Mini-International Neuropsychiatric Interview; XR-BA: extended reality–enhanced behavioral activation.

Figure 2 shows the CONSORT (Consolidated Standards of Reporting Trials) diagram, and Table 1 provides additional participant demographic information.

**Table 1:** Participant demographics (N=26).

Characteristic				XR-BA^a^ (n=13), n (%)		Traditional BA^b^ (n=13), n (%)		Total, n (%)
**Gender**								
	Male			1 (8)		5 (38)		6 (23)
	Female			11 (85)		8 (62)		19 (73)
	Nonbinary or third gender			1 (8)		0 (0)		1 (4)
**Age group (y)**								
	20 to 29			3 (23)		2 (15)		5 (19)
	30 to 39			3 (23)		0 (0)		3 (12)
	40 to 49			1 (8)		2 (15)		3 (12)
	50 to 59			3 (23)		3 (23)		6 (23)
	60 to 69			1 (8)		5 (38)		6 (23)
	70 to 79			2 (15)		1 (8)		3 (12)
**Race or ethnicity**								
	Asian			1 (8)		1 (8)		2 (8)
	Black			0 (0)		1 (8)		1 (4)
	Hispanic or Latino			0 (0)		1 (8)		1 (4)
	Indian			1 (8)		2 (15)		3 (12)
	Mexican			1 (8)		0 (0)		1 (4)
	Non-Hispanic White			10 (77)		8 (62)		18 (69)
**Previous mental health treatment**								
	Yes			12 (92)		12 (92)		24 (92)
	No			1 (8)		1 (8)		2 (8)
**Current mental health treatment**								
	**Yes**			11 (85)		6 (46)		17 (65)
		Psychotherapy only	1 (9)		1 (17)		2 (12)	
		Psychotropic medications only	3 (27)		3 (50)		6 (35)	
		Psychotherapy and medications	7 (64)		2 (33)		9 (53)	
	No			2 (15)		7 (54)		9 (35)
**Previous experience using VR^c^**								
	0 times			9 (69)		9 (69)		18 (69)
	1 to 4 times			3 (23)		3 (23)		6 (23)
	5 to 9 times			1 (8)		1 (8)		2 (8)
	≥10 times			0 (0)		0 (0)		0 (0)
**Purpose of previous VR use**								
	Gaming			3 (75)^d^		2 (50)^d^		5 (62)^e^
	Treatment			0 (0)^d^		0 (0)^d^		0 (0)^e^
	Research			1 (25)^d^		2 (50)^d^		3 (38)^e^

^a^XR-BA: extended reality–enhanced behavioral activation.

^b^BA: behavioral activation.

^c^VR: virtual reality.

^d^n=4.

^e^n=8.

### XR-BA Prototype Feasibility

The completion rates were 77% (10/13) in the XR-BA arm and 85% (11/13) in the traditional BA arm. No participants reported any serious adverse events. The participants in the XR-BA arm used the headset, on average, slightly less than suggested (encouraged a minimum of 12 times), with the completer average being 12 (SD 2.67) and the ITT participant average being 11.18 (SD 3.71). Only 8% (1/13) of the participants did not submit a post-XR questionnaire during 1 week of treatment. This participant reported that she did not use the headset during that week due to being busier than usual with work deadlines and feeling physically ill.

**Figure 3:**
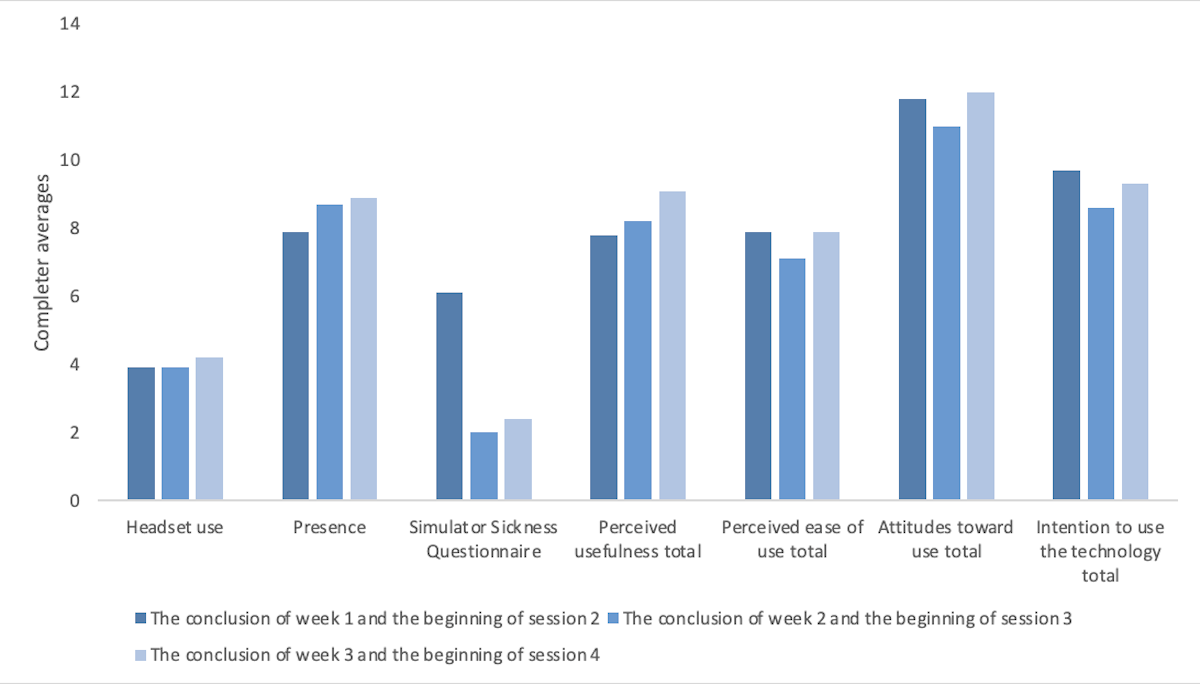
Headset use and post–extended reality questionnaire results by week among protocol completers.

The average total presence rating of the ITT XR-BA participants was 68% (8.1/12; SD 2.5%), whereas the average rating of all the XR-BA completers was 71% (8.5/12; SD 2.2%). The participant who gave the lowest presence rating (3.7/12, 31%, SD 1.15%) shared that “tactile” sensations, such as feeling the sun on her skin, were important to her, and consequently, the VR did not feel immersive. Participants who completed the protocol on average indicated progressively higher levels of presence each subsequent week (Figure 3), although statistical significance was not analyzed.

### XR-BA Acceptability

Overall, the participants who completed the protocol were “neutral” on or “agreed” with the VR treatment being acceptable, with an average rating of 2.8 (SD 0.21; where 2=neutral and 3=agree) on the Likert scale and 71% (37/52) acceptability (Table 2). The participant who gave the lowest acceptability rating (26.7/52, 51%) described that the learning curve of the headset and the discomfort from the weight of the headset made VR less enjoyable. Participants who completed the protocol indicated a higher level of perceived usefulness of VR on average after each subsequent week of use (Figure 3). Between the conclusion of week 1 and the beginning of session 2 and the conclusion of week 3 and the beginning of session 4, participants who completed the protocol reported a lower level of desire to continue using the headset after treatment on average (Figure 3). While participants were provided with the XR activity list (Multimedia Appendix 4), there were certain activities from the list that participants specifically mentioned enjoying, such as YouTube 360° videos (8/13, 62%), *Tripp* (5/13, 38%), *Liminal* (5/13, 38%), *Beat Saber* (2/13, 15%), and *Painting* (2/13, 15%).

**Table 2:** Extended reality–enhanced behavioral activation acceptability.

	Perceived usefulness^a^ (0-12; 3 items), mean (SD)	Perceived ease of use^a^ (0-12; 3 items), mean (SD)	Attitudes toward use^b^ (0-16; 4 items), mean (SD)	Intention to use the technology^a^ (0-12; 3 items), mean (SD)
Completer average	8.4 (2.2)	7.7 (2.6)	11.7 (2.6)	9.2 (1.9)
ITT^c^ average	8.1 (2.3)	7.5 (2.5)	11.1 (3.0)	8.4 (3.3)

^a^Domains comprising the Technology Acceptance Model (higher numbers indicate greater acceptability). Perceived usefulness, perceived ease of use, and intention to use the technology comprised 3 items with a range of 0 (*strongly disagree*) to 4 (*strongly agree*) for each item.

^b^Attitudes toward use comprised 4 items with a range of 0 (*strongly disagree*) to 4 (*strongly agree*) for each item.

^c^ITT: intention to treat.

### XR-BA Tolerability

Physical tolerability was determined using the SSQ. Possible responses for the 16 items ranged from 0 (*no more than usual*) to 3 (*severely more than usual*). Lower numbers indicate greater tolerability. The average overall physical tolerability of those who completed the protocol and the ITT participants was 92% (44/48) and 92% (44.4/48), respectively. *Eyestrain* was the most common symptom of physical intolerability. *Burping* and *increased salivation* were the least common symptoms of physical intolerability, with 8% (1/13) of the participants endorsing burping after week 1 of headset use and 8% (1/13) of the participants endorsing increased salivation after week 1 of headset use. The participant who endorsed relatively higher overall average simulator sickness symptoms (rating of 17/48) compared to other participants experienced most of these symptoms after the first week (rating of 30/48) of headset use. This participant shared that the headset felt uncomfortable and heavy on her head and she experienced symptoms of nausea when she was immersed in any activity that had a quick-moving image. Upon trying other activities within the headset, such as the slower-moving *Liminal* and YouTube 360° videos, this participant’s symptoms reduced to a rating of 4 out of 48. Overall, participants who completed the protocol experienced a decrease in simulator sickness symptoms between the conclusion of weeks 1 and 3 on average (Figure 3).

### Clinical Efficacy

Participants in both study arms showed a 4-point decrease in PHQ-9 scores between sessions 1 and 4, with participants in the XR-BA arm experiencing a 4.4-point decrease and participants in the traditional BA arm experiencing a 3.7-point decrease. There was no significant difference in improvement in PHQ-9 scores between the study arms (t_18.6_=−0.28; *P*=.78; Table 3).

Protocol completers in the XR-BA arm went from an average of moderately severe (15.8, SD 2.86; phone intake) to moderate (session 1: 12.8, SD 3.46; session 2: 10.6, SD 4.14; session 3: 10.5, SD 4.81) to mild (8.4, SD 3.72; session 4) symptoms of depression. The average decrease of 7.4 points on the PHQ-9 between the initial phone screening and session 4 was statistically significant (*t*_9_=4.2; *P*=.002) and represented a clinically significant change in severity level from *moderately severe* to *mild* (>5) [25] (Table 4).

Participants in the traditional BA arm remained at an average of moderately severe (16.0, SD 3.38) between the phone intake and the beginning of session 1 (14.5, SD 3.50), and their symptoms of depression decreased to moderate (10.7, SD 4.63) by session 4. This average decrease of 5.3 points on the PHQ-8 and PHQ-9 between the initial phone screening and session 4 was also statistically significant (t_10_=2.88; *P*=.02) and represented a change in clinical severity from *moderately severe* to *moderate* (Table 5).

There was a significant decrease in PHQ-9 scores between the phone intake and the beginning of session 1 in the XR-BA group (t_11_=2.6; *P*=.03) but not in the traditional BA group (t_11_=1.4; *P*=.20). These results indicate that participants in the XR-BA arm showed a significant decrease in PHQ-9 scores even before the treatment began.

To determine whether the participants showed a further statistically significant decrease in PHQ-9 scores between the beginning of sessions 1 and 4, paired-sample *t* tests were run. These results illustrated that participants in both the XR-BA (*t*_9_=2.5; *P*=.04) and the traditional BA (t_10_=2.3; *P*=.04) arms experienced a significant decrease in PHQ-9 scores between the start and end of the study (Tables 4 and 5).

**Table 3:** Independent-sample *t* test—extended reality–enhanced behavioral activation (XR-BA) versus traditional behavioral activation (BA) and test of significance of the difference between the 2 groups.

	XR-BA (n=10), mean (SD)	Traditional BA (n=11), mean (SD)	*t* test (*df*)	2-sided *P* value
Difference between PHQ-8^a^ at intake and PHQ-9^b^ at session 4	7.40 (5.54)	5.27 (6.07)	−0.84 (19)	.41
Difference between PHQ-9 at session 1 and PHQ-9 at session 4	4.40 (5.66)	3.73 (5.37)	−0.28 (19)	.78

^a^PHQ-8: Patient Health Questionnaire–8.

^b^PHQ-9: Patient Health Questionnaire–9.

**Table 4:** Paired-sample *t* test (2-tailed)—testing the significance of extended reality–enhanced behavioral activation participants’ average Patient Health Questionnaire scores between various time points (n=13).

	Pretest assessment		Posttest assessment		*t* test (*df*)	2-sided *P* value
	Participants, n (%)	Values, mean (SD)	Participants, n (%)	Values, mean (SD)		
PHQ-8^a^ at phone intake and PHQ-9^b^ at session 4	10 (77)	15.80 (2.86)	10 (77)	8.40 (3.72)	4.22 (9)	.002
PHQ-8 at phone intake and PHQ-9 at session 1	12 (92)	15.67 (2.90)	12 (92)	13.25 (3.82)	2.59 (11)	.03
PHQ-9 at session 1 and PHQ-9 at session 4	10 (77)	12.80 (3.46)	10 (77)	8.40 (3.72)	2.46 (9)	.04
PHQ-9 at session 1 and PHQ-9 at session 2	11 (85)	13.45 (3.93)	11 (85)	11.55 (5.03)	1.70 (10)	.12
PHQ-9 at session 2 and PHQ-9 at session 3	10 (77)	10.60 (4.14)	10 (77)	10.50 (4.81)	0.07 (9)	.95
PHQ-9 at session 3 and PHQ-9 at session 4	10 (77)	10.50 (4.81)	10 (77)	8.40 (3.72)	1.41 (9)	.19

^a^PHQ-8: Patient Health Questionnaire–8.

^b^PHQ-9: Patient Health Questionnaire–9.

**Table 5:** Paired-sample *t* test (2-tailed)—testing the significance of traditional behavioral activation participants’ average Patient Health Questionnaire scores between various time points (n=13).

	Pretest assessment		Posttest assessment		*t* test (*df*)	2-sided *P* value
	Participants, n (%)	Values, mean (SD)	Participants, n (%)	Values, mean (SD)		
PHQ-8^a^ at phone intake and PHQ-9^b^ at session 4	11 (85)	16.00 (3.38)	11 (85)	10.73 (4.63)	2.88 (10)	.02
PHQ-8 at phone intake and PHQ-9 at session 1	12 (92)	16.00 (3.22)	12 (92)	14.75 (3.49)	1.37 (11)	.20
PHQ-9 at session 1 and PHQ-9 at session 4	11 (85)	14.45 (3.50)	11 (85)	10.73 (4.63)	2.30 (10)	.04
PHQ-9 at session 1 and PHQ-9 at session 2	11 (85)	14.45 (3.50)	11 (85)	12.09 (3.59)	2.95 (10)	.01
PHQ-9 at session 2 and PHQ-9 at session 3	11 (85)	12.09 (3.59)	11 (85)	11.82 (2.64)	0.25 (10)	.81
PHQ-9 at session 3 and PHQ-9 at session 4	11 (85)	11.82 (2.64)	11 (85)	10.73 (4.63)	1.17 (10)	.27

^a^PHQ-8: Patient Health Questionnaire–8.

^b^PHQ-9: Patient Health Questionnaire–9.

## Discussion

### Feasibility

The results of this study provide evidence that XR-BA in this MDD telehealth treatment setting was a safe, feasible, tolerable, and acceptable modification to a brief BA protocol. The attrition rate of 23% (3/13) of the participants in the XR-BA arm of the study is comparable with that of other VR studies [26,27], lower than that of many RCTs of internet-based interventions for depression [28], and lower than that of a small-sample pilot RCT exploring exercise as a treatment for depression [29]. Importantly, no participant in the XR-BA treatment arm dropped out of the study because of serious adverse events, and no serious adverse events were reported throughout the study.

While participants in the previous VR-BA study completed on average more VR activities than recommended, the participants in this study did not meet their total recommended headset use of ≥4 activities each week. This was due to many participants reporting that the headset was difficult to use and it feeling like an overwhelming task to learn. Participants remarked that they would have used the headset more often if they had increased familiarity. In this vein, participants reported that the headset became more enjoyable and useful over time, which aligns with research that states that the easier to use the device, the more acceptable it is to users [30]. When working with people unfamiliar with VR, future prototypes of VR-BA may want to opt for designs that allow for simplicity, preloaded experiences, decreased choices, and rapid onboarding skill acquisition.

Participants noted several barriers and XR challenges that may have impacted their attempts to use this modality for BA and mood improvement. The learning curve for using the headset device was surprisingly burdensome. Our previous VR-BA prototype chose a simpler, less immersive headset preloaded with activity choices, but this study chose to use a more immersive and interactive headset with higher quality and range of choices of pleasant and mastery activities. This increase in variety and autonomy to simulate traditional BA came with an increasing cost to the user, with each novel experience entailing unique and new technical XR challenges and requiring new skills. This observation aligns with research indicating that it is important to learn *how* to use VR before learning *in* VR [31]. Spending time teaching participants how to use the XR headset was contraindicated in a research study due to the creation of a confound when compared to traditional BA. Yet, in practice and outside of clinical trials, it may be necessary to do so at this time when technical onboarding to commercial headsets is still complex and challenging for the average person. However, the challenges of onboarding may just as likely provide an opportunity to engage in a mastery or pleasant activity when struggling and finally gaining access to the XR headset and may actually attenuate BA with the focus on this acquisition of onboarding skills.

One participant noted that the ability to choose any activity on the headset led to “decision paralysis,” an interesting juxtaposition to the previous study, which had a limited selection of 37 preselected videos and where feedback stated a desire to have more activity options. While activity ideas were provided, when using XR for activity engagement, it may be helpful to provide an even more detailed database of activity options similar to the list of adult pleasant activities [32].

Considering participant feedback from the previous study that noted that the requirement to complete a post-VR questionnaire after each use was a hindrance and burden, this study only asked participants to complete 1 post-XR questionnaire a week. While only 20% (1/5) of the participants in the previous study completed a post-VR questionnaire for each VR activity, all participants in this study (13/13, 100%) completed a post-XR questionnaire during the weeks in which they used the device. Participants in this study subsequently did not comment on the administrative burden of completing the post-XR questionnaire; however, they did acknowledge that having all the tracking and scheduling accessible via the web or through an app would make it more convenient for them to remember and complete all the required tasks.

Participants in this study rated their presence as higher on average than participants in the previous study, a finding that is consistent with research suggesting that achieving a strong sense of presence is more influenced by interactivity than by realism [33]. Participants noted feeling so present while using the headset that they made comments such as the following: “[it was] good to be able to go elsewhere [in VR] since I don’t have a car,” “it is nice to be able to take a break from my kids and be present at home, but not be,” and “I was so immersed in the VR that I lost track of time.” In addition, presence ratings increased week to week on average, consistent with participant reports that the more familiar they became with the device, the more immersed they felt.

The acceptability ratings in this study were comparatively lower than those recorded for the device used in the previous study. Nevertheless, a noteworthy observation from this study is that acceptance levels in the domains of *Perceived usefulness* and *Attitudes toward use* exhibited an average increase between the conclusion of week 1 and the beginning of session 2 and the conclusion of week 3 and the beginning of session 4. It would be intriguing to extend the study timeline and ascertain whether this trend of escalating acceptance continues, potentially surpassing the ratings for the simpler headset. It would be equally fascinating to explore whether the gradual rise in acceptance over time corresponds to more substantial improvements in mood over the same period. This is particularly relevant considering that some participants mentioned that they would have used the device more frequently if they had not perceived the learning curve as a hindrance. Furthermore, participants qualitatively indicated that the *Intention to use the technology* rating was lower given the cost and lack of affordability of the Meta Quest 2 headset.

The participants rated the protocol as largely physically tolerable, and no participants dropped out because of adverse effects. While the ratings of physical tolerability were the same (92%-93%) between the 2 studies, the participants in this study qualitatively reported more simulator sickness. Participants particularly noted that they found the Meta Quest 2 headset itself to be “heavy” and “uncomfortable” on their faces. In addition, consistent with the research on simulator sickness, participants noted that they experienced more symptoms of simulator sickness while partaking in activities with a faster-moving image compared to those with a slower-moving image [34,35]. However, also aligned with previous research, participants quantitatively and qualitatively reported a habituation effect where their simulator sickness symptoms largely decreased over time [35]. All participants reported that their symptoms were quickly resolved upon removal of the headset and did not persist.

While this study expanded upon the previous study by increasing the sample size and using a more immersive, interactive headset that offered a wider range of activity options, it would be interesting to conduct a similar study that also uses a mobile app to decrease the administrative burden for providers and patients and streamline the homework process. It is postulated that the focus on homework in BA is essential for successful treatment outcomes. Research has demonstrated that homework completion is significantly related to a decrease in symptoms [36]. Specifically, the behavioral task of completing pleasant activities contributed most strongly to decreasing symptoms of depression [36]. Hence, addressing barriers to completing homework tasks is pivotal for optimizing treatment results.

Given that participants in the previous study noted decreased headset use due to administrative constraints and that participants in this study independently expressed the value of a tracking and reminder app for homework compliance, the next crucial phase involves evaluating whether implementing a mobile app that consolidates scheduling and activity tracking can enhance homework completion rates. This, in turn, could potentially lead to more accurate and consistent homework adherence, thereby further reducing depressive symptoms and enhancing mood, ultimately maximizing the effectiveness of treatment outcomes.

Finally, this study solely used XR as a method of engaging in BA. In subsequent studies, it would be interesting to conduct the therapy in VR for both arms rather than over Zoom. Given that telehealth has become increasingly popular, it would be fascinating to note the feasibility, acceptability, and tolerability of conducting the entire session in VR. It would also be interesting to measure how this may affect the effectiveness of the intervention.

### Efficacy

The XR-BA protocol was found to be noninferior to a brief BA protocol for MDD in this small study. Participants in both the traditional and XR-BA arms experienced a statistically significant reduction in depression symptoms between the initial phone screening and session 4 and between sessions 1 and 4, as well as significant reductions in clinical severity between the initial phone screening and session 4.

Only the XR-BA arm showed a statistically significant decrease in symptoms between the phone screening and session 1. Given the unblinded nature of this study, this is not surprising. These results may indicate that participants in the XR-BA arm had an enhanced expectancy or placebo effect due to the novelty or implicit beliefs surrounding technology and mental health treatment. The novelty of the treatment and anticipation that it would be helpful may have led to increased levels of hope and a decrease in depressive symptoms [37,38]. It was observed that the participants who learned that they were randomized into the XR-BA arm expressed more excitement than those who were randomized into the traditional BA arm.

While our previous study suggested the possibility of a greater reduction in symptoms of depression among participants in the VR-BA arm compared to the traditional BA arm, this study did not demonstrate any such superiority as symptom reduction was not statistically or clinically different between the groups. The noninferiority of XR-BA may be attributed to both the positives and negatives of using VR, as noted by participants. Similar to the previous study, participants shared that they found VR to be “novel,” using VR showed them that they could enjoy activities again, and VR inspired them to engage in real-life activities. The latter fact was true among both participants who found VR to be a positive experience (ie, watching a YouTube 360° video of a beach inspired them to visit the beach in person) and a participant who did not enjoy VR because of preferences for tactile experiences and consequently made an increased effort to go outside to feel the sun on their skin. Many participants also noted that the XR-BA helped improve their mood by taking them to a new place in an immersive way, thereby increasing their attention and decreasing distraction, allowing for a fully mindful experience in the present moment.

The finding that XR-BA was as efficacious in reducing symptoms of MDD as a brief traditional BA protocol is critical. Patients can use VR to improve their mood if they encounter barriers to engaging in activities IRL. Participants commented that “VR is easier and more convenient than having to go places,” “I have been able to visit a few places I have always wanted to travel, so I noticed being so absorbed [by the places],” “VR has a larger realm of possibilities. In the real world I need to check hours [that events are occurring/open] and the weather,” and “I would recommend [using VR] to a friend if they didn’t want to do therapy,” which qualitatively supports the notion that VR can help decrease barriers to in-person activity engagement. These statements further corroborate the previous study’s suggestion that clinicians may be justified in using VR as a first step in BA for patients who may not have access, motivation, or desire to engage in activities IRL.

### Limitations

This study aimed to recruit and enroll 40 participants, and recruitment took place remotely via Zoom-delivered telehealth sessions between December 19, 2022, and July 24, 2023. The study ended recruitment in July 2023 given that the necessary number of participants to yield a powered result had been enrolled. Although many of the enumerated findings are promising, this study has several limitations. First, the quantitative and qualitative measures were subjective and completed by the participants. Participants in both the XR-BA and traditional BA arms self-reported their completed activity and mood scores, which may have introduced inaccurate reporting. Specifically, there were no objective measures used to evaluate XR experiences, which resulted in participant self-report of the activities chosen and length of time in XR, which many participants did not document in the moment, leading to potentially inaccurate reporting. In addition, although the PHQ-9 is a standard self-report measure, the questions were read aloud for participants to answer rather than being delivered in a standard written format. This method may have resulted in less accurate reporting if the participants felt inclined to respond in a certain way.

Furthermore, as there were no official follow-ups, it is unknown whether the mood gains that the participants reported were long-lasting. This study had a relatively short duration. As mentioned previously, participants remarked on the learning curve of the headset, and both qualitative and quantitative data illustrated that the headset became more acceptable and tolerable each week. Thus, in a longer trial, participants may experience greater mood gains as they become more familiar with the headset. In addition, a participant in the XR-BA arm of the study was unable to use the headset between sessions 2 and 3 owing to both a heavy work week and being physically ill. This participant expressed sadness about this outcome and a desire to expand the study timeline to have more time with the headset. Furthermore, the study’s short duration may have led to mood changes due to factors external to the study, such as a relatively heavy or light work week or an illness. Finally, many participants expressed that there were few free trials or options within the XR headset. Some participants reported that they would be more willing to purchase activities if they were able to keep the headset or if the study were longer so that they had more time with their purchase. Overall, participants in both study arms expressed a desire to lengthen the study timeline and noted that the 3-week, 4-session protocol felt too short.

Another limitation was the nonblinded nature of the study. Participants randomized into the XR-BA arm expressed greater excitement than those randomized into the traditional BA arm, which may have led to an initially greater decrease in depressive symptoms on the former. Further studies should invest in a system of double blinding to confirm these results.

Finally, as in our previous study, recruitment was a large obstacle. Although the goal was to randomize 40 participants with MDD into either of the study arms, only 26 participants were randomized because other potential participants were excluded based on ineligibility, declining to participate, or being lost to follow-up. It is notable that other VR and depression studies have had similar or smaller sample sizes [39,40]. It is important to recognize that this could underscore an inherent challenge in depression studies, where health state and conditional altruism are large contributing factors to participation interest [41]. In addition, the small sample size hindered our ability to address other interesting research questions, such as whether different subtypes or severity of MDD would yield different effects from the treatment. For example, would individuals with more severe MDD respond better or worse to XR-BA than those with a milder case compared to traditional BA? These are questions that would need to be answered in a study with a larger sample size. Moreover, given the diverse nature of the disorder, the findings might not universally apply to all those dealing with symptoms of depression.

### Conclusions

The findings of this study support our previous report that using XR as a substitute for IRL pleasant and mastery activities in a brief BA protocol for individuals diagnosed with MDD is feasible, acceptable, and tolerable. This remained true even when using a more difficult and interactive headset that posed technical and physical challenges.

This study also expanded on our feasibility trial to perform the first known efficacy trial of XR-BA. This study demonstrated that XR-BA may not be inferior to traditional BA as it was equally and statistically efficacious in improving symptoms of depression in an MDD sample as measured using the PHQ-9. It also suggested that XR-BA may have enhanced the placebo or expectation effects of BA treatment.

The results of this study demonstrate that it may not be unreasonable for clinicians to suggest the use of VR-simulated pleasant activities to patients when delivering BA as VR-simulated pleasant activities may offer solutions to some of the common problems and barriers encountered when using BA. When deciding on a clinical approach, professionals may need to weigh the advantages and disadvantages of using simpler versus complex headsets. Given that this study and the previous VR-BA study both illustrate clinical effectiveness and that the feedback on the previous study’s preloaded headset was more favorable compared with this study’s software-agnostic Meta Quest 2 headset, it could be concluded that a simpler device would be preferred by patients at this time. Furthermore, despite this study providing the opportunity for participants to choose among a multitude of VR options, the most commonly chosen VR activity was YouTube 360° videos largely given its simplicity and lack of cost. Regardless of the hardware or software specifications, this study supports the notion that the use of VR may enhance mood in those living with MDD when used in conjunction with individual therapy delivering BA principles and protocols. More research on the implementation of such an approach is needed to understand how to most effectively leverage this technology in depressive disorders.

Future and more extensive controlled studies may want to explore further whether XR can increase expectation or placebo effects during MDD treatments or have other enhancing qualities to the delivery of BA for MDD.

## Appendix

Multimedia Appendix 1 Telephone screening questions.

Multimedia Appendix 2 Consent form.

Multimedia Appendix 3 Demographic questionnaire.

Multimedia Appendix 4 Extended reality activity list.

Multimedia Appendix 5 Post–extended reality questionnaire.

Multimedia Appendix 6 CONSORT-EHEALTH (Consolidated Standards of Reporting Trials of Electronic and Mobile Health Applications and Online Telehealth) V 1.6.1 checklist.

## Abbreviations

AMOS: analysis of moment structures
BA: behavioral activation
CONSORT: Consolidated Standards of Reporting Trials
EBP: evidence-based psychotherapy
IRL: in real life
ITT: intention-to-treat
MDD: major depressive disorder
PHQ-8: Patient Health Questionnaire–8
PHQ-9: Patient Health Questionnaire–9
RCT: randomized controlled trial
SEM: structural equation modeling
SSQ: Simulator Sickness Questionnaire
VR: virtual reality
VR-BA: virtual reality–enhanced behavioral activation
XR: extended reality
XR-BA: extended reality–enhanced behavioral activation

## Data Availability

The data sets generated and analyzed during this study are available from the corresponding author on reasonable request.
