# Supplementary material for: Examining the Efficacy of Extended Reality–Enhanced Behavioral Activation for Adults With Major Depressive Disorder: Randomized Controlled Trial
Source: JMIR Ment Health. 2024 Apr 15;11:e52326. doi: 10.2196/52326 (PMC11058556; doi:10.2196/52326)
Supplement: Multimedia Appendix 1 [file mental_v11i1e52326_app1.docx]

Telephone Screen Questions

1. What is your name?
2. When is your birthday?
3. What is the language you feel most comfortable speaking?
4. Have you ever been diagnosed with psychosis or bipolar disorder?
5. Have you experienced any seizures in the past 6 months?
6. If yes, are your seizures currently being treated?
7. Are you currently seeing a psychotherapist?
   1. If yes, how long have you been seeing them?
8. Have you changed (or are you planning to change) psychotropic medications within two months?

Ask PHQ-8
