# Supplementary material for: Examining the Efficacy of Extended Reality–Enhanced Behavioral Activation for Adults With Major Depressive Disorder: Randomized Controlled Trial
Source: JMIR Ment Health. 2024 Apr 15;11:e52326. doi: 10.2196/52326 (PMC11058556; doi:10.2196/52326)
Supplement: Multimedia Appendix 2 [file mental_v11i1e52326_app2.docx]

Are you participating in any other research studies? Yes No

**PURPOSE OF RESEARCH**

This research study is looking for 40 adults with Major Depressive Disorder (MDD). Stanford University expects to enroll all 40 research study participants. You are invited to participate in a research study on the use of behavioral activation or extended reality (XR) behavioral activation therapy for MDD. You were selected as a possible participant in this study because you meet criteria for MDD. The purpose of the study is to learn more about behavioral activation as a treatment for MDD. The XR device is investigational and is not FDA approved for use as described in this study.

If you decide to terminate your participation in this study, you should notify Margot Paul at 650-736-1569.

**VOLUNTARY PARTICIPATION**

Your participation in this study is entirely voluntary. Your decision not to participate will not have any negative effect on you or your medical care. You can decide to participate now but withdraw your consent later and stop being in the study without any loss of benefits or medical care to which you are entitled.

**DURATION OF STUDY INVOLVEMENT**

If you are randomized to the XR behavioral activation or behavioral activation as usual groups, your participation in this experiment will take less than eight hours total. You will receive 4 weekly sessions of approximately 50 minutes in duration over 3 weeks. This will include approximately 40 minutes of behavioral activation therapy and 10 minutes of filling out surveys each week. Additionally, you will be asked to perform the behavioral activation activities you identified at home. These time estimates do not include your specific travel time from home to Stanford.

**PROCEDURES**

If you decide to participate, you will be randomized to receive XR behavioral activation

or behavioral activation as usual. Your study group will be randomly selected (like flipping a coin). You will have a 50% chance of being randomized into any one group.

Behavioral activation is defined as identifying real-life pleasant and/or mastery activities, scheduling them into your calendar, and completing the identified activities during the week, which is behavioral activation treatment as usual. The XR behavioral activation will consist of completing identified activities solely in the XR headset. In the behavioral activation as usual and XR behavioral activation cases you will receive 4 one-hour weekly sessions. You will also be asked to take several surveys and provide feedback. You will be asked to complete a weekly 9-question depression screener (PHQ-9). Due to the 9th question asking about suicidality, the examiner will ask your current location before the screener is administered each week. If you endorse question 9, a risk assessment will be completed in real-time and may include creating a safety plan, or in more extreme circumstances, calling the police for a welfare check. Audio and video taping may occur while you participate, but that data will not be linked to your personal information, and the data will be destroyed after it has been coded and transcribed.

It is possible that, based on information gained from this study, the researchers may have serious concerns (relating to matters such as severe depression, physical abuse, etc.) about your health and/or safety; in such a case, the researchers may contact you and provide a referral for your care.

I give consent to be audiotaped and videotaped during this study:

Please initial: _Yes No

**PARTICIPANT RESPONSIBILITIES:**

As a participant, your responsibilities include:

- Follow the instructions of the Protocol Director and study staff.
- Keep your study appointments. If it is necessary to miss an appointment, please contact the Protocol Director or research study staff to reschedule as soon as you know you will miss the appointment.
- Tell the Protocol Director or research study staff about any side effects, doctor visits, or hospitalizations that you may have.
- Complete your questionnaires as instructed.
- Ask questions as you think of them.
- Tell the Protocol Director or research staff if you change your mind about staying in the study.

**WITHDRAWAL FROM STUDY**

If you first agree to participate and then you change your mind, you are free to withdraw your consent and discontinue your participation at any time. Your decision will not affect your ability to receive medical care and you will not lose any benefits to which you would otherwise be entitled.

If you decide to withdraw your consent to participate in this study, you should notify Margot Paul at 650-736-1569.

If you withdraw from the study and you acquired an Oculus Quest 2 headset for your XR treatment, you are expected to return it to Margot Paul.

The Protocol Director may also withdraw you from the study without your consent for one or more of the following reasons:

- Failure to follow the instructions of the Protocol Director and study staff.
- Unanticipated circumstances.
- Newly meeting exclusion criteria.

**POSSIBLE RISKS, DISCOMFORTS, AND INCONVENIENCES**

There are risks, discomforts, and inconveniences associated with any research study. These deserve careful thought. Uncomfortable emotions, worsening of your condition, or disappointment from lack of improvement are possible.

Inconveniences that can be expected in this study include weekly travel to Stanford Hospital and Clinics. As is the case in standard clinical care, there is a risk that your medical information may be disclosed to a health oversight agency or to law enforcement officials in certain limited circumstances without your authorization, such as if you are at risk of harming yourself or others.

You should talk with the Protocol Director if you have any questions.

The risks associated with this study are no more extreme than every-day activities such as virtual rides in an amusement park. During participation, you could begin to experience one or more of the symptoms of “cybersickness” (similar to motion sickness): disorientation, dizziness, disturbance of eye-hand coordination, eyestrain, headache, lightheadedness, nausea, and sweating. If you have any of these symptoms, it is possible that one or more of these could last for some time after participation.

When you are participating in this research study, if there is any change in the way you are feeling, let the experimenter know immediately and he/she will end the experiment.

In addition, you are free to terminate the experiment at any time. We will ask you to remain in the experimental situation until any side-effects have subsided. If you experience any lasting symptoms or have other comments to make, please contact Margot Paul at 650-736-1569.

**POTENTIAL BENEFITS**

The benefits which may reasonably be expected to result from this study and standard treatment which are improvement in symptoms and quality of life. In addition, there is the possibility of remission of symptoms, improvement in mood and comorbid psychiatric symptoms, and increased sense of well-being. You may also benefit from the meaning and value you gain from being a part of a scientific study and the possibility of helping others suffering with similar illnesses. We cannot and do not guarantee or promise that you will receive any benefits from this study. Your decision whether or not to participate in this study will not affect your employment/medical care.

**ALTERNATIVES**

The alternative procedures that may be advantageous would include not to participate in this study and remaining on the clinic waitlist and beginning a course of therapy decided by your provider. Another alternative would be to meet with a psychiatrist and discuss medication alternatives. It is important to discuss potential risks and benefits of the alternatives with a physician.

If you have read this form and have decided to participate in this project, please understand your participation is voluntary and you have the right to withdraw your consent or discontinue participation at any time without penalty or loss of benefits to which you are otherwise entitled.

You should not feel obligated to agree to participate. Your questions should be answered clearly and to your satisfaction. If you decide not to participate, tell the Protocol Director. Your decision not to participate will not have any negative effect on you or your medical care.

You will be told of any important new information that is learned during the course of this research study, which might affect your condition or your willingness to continue participation in this study.

**Clinical Trials.gov**

A description of this clinical trial will be available on [*http://www.ClinicalTrials.gov*](http://www.clinicaltrials.gov/)*,* as required by U.S. Law. This Web site will not include information that can identify you. At most, the Web site will include a summary of the results. You can search this Web site at any time.

**CONFIDENTIALITY**

The results of this research study may be presented at scientific or medical meetings or published in scientific journals. Your identity and/or your personal health information will not be disclosed except as authorized by you or as required by law**.** However, there is always some risk that even de-identified information might be re-identified. You have the right to refuse to answer particular questions.

Patient information may be provided to Federal and other regulatory agencies as required. The Food and Drug Administration (FDA), for example, may inspect research records and learn your identity if this study falls within its jurisdiction.

One of the purposes of this research study is to obtain data or information on the safety and effectiveness of Extended Reality (XR) Behavioral Activation Therapy for Major Depressive Disorder (MDD); the results will be provided to the sponsor, the Food and Drug Administration and other federal and regulatory agencies as required.

Identifiers might be removed from identifiable private information and/or identifiable specimens and, after such removal, the information and/or specimens could be used for future research studies or distributed to another investigator for future research studies without additional informed consent from you.

**Authorization To Use Your Health Information For Research Purposes**

Because information about you and your health is personal and private, it generally cannot be used in this research study without your written authorization. If you sign this form, it will provide that authorization. The form is intended to inform you about how your health information will be used or disclosed in the study. Your information will only be used in accordance with this authorization form and the informed consent form and as required or allowed by law. Please read it carefully before signing it.

**What is the purpose of this research study and how will my health information be utilized in the study?**

The purpose of this study is to investigate the feasibility, acceptability, safety and possible efficacy of delivering behavioral activation therapy via extended reality for adults diagnosed with Major Depressive Disorder (MDD).

At the end of the study, information about you and your experience may be used to help direct future research and it may appear in a publication.

**Do I have to sign this authorization form?**

You do not have to sign this authorization form. But if you do not, you will not be able to participate in this research study. Signing the form is not a condition for receiving any medical care outside the study.

**If I sign, can I revoke it or withdraw from the research later?**

If you decide to participate, you are free to withdraw your authorization regarding the use and disclosure of your health information (and to discontinue any other participation in the study) at any time. After any revocation, your health information will no longer be used or disclosed in the study, except to the extent that the law allows us to continue using your information (e.g., necessary to maintain integrity of research). If you wish to revoke your authorization for the research use or disclosure of your health information in this study, you must write to: Margot Paul, Psy.D. 401 Quarry Road, Stanford, CA 94304.

**What Personal Information Will Be Obtained, Used or Disclosed?**

Your health information related to this study may be used or disclosed in connection with this research study, including, but not limited to your name, telephone numbers, street address, city, county, precinct, zip code, all elements of dates (related to an individual, including birth date, admission date, discharge date), fax numbers, medical record numbers, health plan beneficiary numbers, biometric identifiers, health history, descriptive of demographics, and responses to questionnaires and worksheets, video/audio of therapy sessions, and therapy notes.

**Who May Use or Disclose the Information?**

The following parties are authorized to use and/or disclose your health information in connection with this research study:

- The Protocol Director, Margot Paul, Psy.D.
- The Stanford University Administrative Panel on Human Subjects in Medical Research and any other unit of Stanford University as necessary
- Research Staff

**Who May Receive or Use the Information?**

The parties listed in the preceding paragraph may disclose your health information to the following persons and organizations for their use in connection with this research study:

- The Office for Human Research Protections in the U.S. Department of Health and Human Services
- The Food and Drug Administration

Your information may be re-disclosed by the recipients described above, if they are not required by law to protect the privacy of the information.

**When will my authorization expire?**

Your authorization for the use and/or disclosure of your health information will end on December 31, 2100 or when the research project ends, whichever is earlier.

**Will access to my medical record be limited during the study?**

To maintain the integrity of this research study, you may not have access to any health information developed as part of this study until it is completed. At that point, you would have access to such health information if it was used to make a medical or billing decision about you (e.g., if included in your official medical record).

____________________________________ ______________

Signature of Adult Participant Date

________________________________________

Print Name of Adult Participant

**FINANCIAL CONSIDERATIONS**

You will not be paid to participate in this research study. There is no cost to you for participating in this study, other than basic expenses like transportation and the personal time it will take to come to all of the study visits.

Wu Tsai Neuroscience Institute is providing financial support for this study.

**COMPENSATION for Research-Related Injury**

All forms of medical diagnosis and treatment – whether routine or experimental

- involve some risk of injury. In spite of all precautions, you might develop medical complications from participating in this study. If such complications arise, the Protocol Director and the research study staff will assist you in obtaining appropriate medical treatment. In the event that you have an injury or illness that is directly caused by your participation in this study, reimbursement for all related costs of care first will be sought from your insurer, managed care plan, or other benefits program. **You will be responsible for any associated co-payments or deductibles as required by your insurance.**

If costs of care related to such an injury are not covered by your insurer, managed care plan or other benefits program, you may be responsible for these costs. If you are unable to pay for such costs, the Protocol Director will assist you in applying for supplemental benefits and explain how to apply for patient financial assistance from the hospital.

You do not waive any liability rights for personal injury by signing this form.

**CONTACT INFORMATION**

Questions, Concerns, or Complaints: If you have any questions, concerns or complaints about this research study, its procedures, risks and benefits, or alternative courses of treatment, you should ask the Protocol Director, Margot Paul. You may contact her now or later at 650-736-1569. You should also contact her if you feel you have been hurt by being a part of this study.

Alternate Contact: If you cannot reach the Protocol Director, please contact Kim Bullock at 650-714-1459.

Independent Contact: If you are not satisfied with how this study is being conducted, or if you have any concerns, complaints, or general questions about the research or your rights as a participant, please contact the Stanford Institutional Review Board (IRB) to speak to someone independent of the research team at (650)-723-5244 or toll free at 1-866-680-2906. You can also write to the Stanford IRB, Stanford University, 1705 El Camino Real, Palo Alto, CA 94306.

**EXPERIMENTAL SUBJECT’S BILL OF RIGHTS**

As a research participant you have the following rights. These rights include but are not limited to the participant's right to:

- - be informed of the nature and purpose of the experiment;
  - be given an explanation of the procedures to be followed in the medical experiment, and any drug or device to be utilized;
  - be given a description of any attendant discomforts and risks reasonably to be expected;
  - be given an explanation of any benefits to the subject reasonably to be expected, if applicable;
  - be given a disclosure of any appropriate alternatives, drugs or devices that might be advantageous to the subject, their relative risks and benefits;
  - be informed of the avenues of medical treatment, if any available to the subject after the experiment if complications should arise;
  - be given an opportunity to ask questions concerning the experiment or the procedures involved;
  - be instructed that consent to participate in the medical experiment may be withdrawn at any time and the subject may discontinue participation without prejudice;
  - be given a copy of the signed and dated consent form; and
  - be given the opportunity to decide to consent or not to consent to a medical experiment without the intervention of any element of force, fraud, deceit, duress, coercion or undue influence on the subject's decision.

May we contact you about future studies that may be of interest to you? Yes ☐ No ☐

Signing your name means you agree to be in this study and that you will receive a copy of this signed and dated consent form.

_

Signature of Adult Participant Date

_

Print Name of Adult Participant
