# Supplementary material for: Examining the Efficacy of Extended Reality–Enhanced Behavioral Activation for Adults With Major Depressive Disorder: Randomized Controlled Trial
Source: JMIR Ment Health. 2024 Apr 15;11:e52326. doi: 10.2196/52326 (PMC11058556; doi:10.2196/52326)
Supplement: Multimedia Appendix 4 [file mental_v11i1e52326_app4.docx]

**XR Activity List**

Check the ones you are willing to do, and then add any other activities you can think of:

Gaming

- A Fisherman’s Tale
  - Puzzle and adventure game
- Demeo
  - Play a magical VR board game
- Echo VR
  - Battle robots in zero gravity
- Eleven: Table Tennis
  - Play table tennis
- Space Pirate Trainer DX
  - Enter the arcade game
- The Climb
  - Rock climb in VR
- The Room VR: A Dark Matter
  - Use clues to solve puzzles
- Ultrawings 2
  - Fly aircrafts and win missions
- Unplugged
  - Play some air guitar
- Walkabout Mini Golf
  - Play mini golf alone or with friends
- Others
  - Choose hundreds of others from the app store

Fitness and Wellness

- Beat Saber
  - Slice blocks to the beat
- Liminal
  - Experience calm, energy, and awe on this platform
- Tripp
  - Explore amazing visuals while you meditate
- Dance Central
  - Dance and groove to the music
- Fit XR
  - Join on demand workout classes
- Holofit
  - Workout in VR
- OhShape VR
  - Move your body with rhythm
- Smash Drums
  - Drum in VR
- Supernatural
  - Workout in VR
- Thrill of the Fight
  - Try your hand at VR boxing
- VZ Fit
  - Workout in VR while exploring the world (can use a stationary bike)
- Others
  - Choose hundreds of others from the app store

Social (Note: Due to the fact that other users are able to interact with you in these environments, there are inherent risks such as being exposed to profanity or other inappropriate language/remarks.)

- Altspace
  - Create and/or attend live events (i.e., concerts, conferences, comedy shows, festivals, etc).
  - EvolVR: partake in daily meditation groups
- Bigscreen
  - Watch movies with other people in VR
- Couch
  - Watch YouTube with other people in VR
- Engage VR
  - Attend or host conferences, meetings, or classes
  - Build your own content or explore what is already out there
- Horizon Venues
  - Attend a concert or game
- Horizon Worlds
  - Join block parties and events around the world
- Rec Room
  - Build, play games, and chat with people from around the world
- vTime XR
  - Meet chat, share photos, and watch content with other people around the world
- VRChat
  - Embody an avatar to play games (i.e., mini golf, escape rooms, karaoke, etc.) and chat with people from around the world
- Others
  - Choose hundreds of others from the app store

Productivity and Education

- Anne Frank House
  - Learn about Anne Frank
- Google Tilt Brush
  - Paint in VR
- Mission: ISS
  - Simulate being in space and learn how to navigate zero gravity
- Mondly
  - Practice languages in VR
- National Geographic Explore VR
  - Visit some of the world’s most iconic sites
- Noda
  - Build and share 3D mental models
- Ocean Rift
  - Explore and learn about the ocean
- Painting VR
  - Paint in VR
- Traveling While Black
  - Learn about the history of the restriction of movement for Black individuals
- Tribe XR
  - Become a DJ in VR
- Others
  - Choose hundreds of others from the app store

VR 360

- Check the Views at Whistler, Just Scroll Around
  - Ski and observe the beautiful winter sights of Whistler
- Hamilton: An American Musical 360
  - Practice “Wait for It” with the cast of Hamilton
- National Geographic: “As it is”
  - Explore the Grand Canyon
- National Geographic: Expedition Everest: The Science
  - Learn about climate change in Everest
- National Geographic: Free Solo 360
  - Climb Yosemite’s famous El Capitan with Alex Honnold
- National Geographic: Journey into the Deep Sea
  - Explore the oceans of Palau
- National Geographic: Lions 360
  - Learn about African lions
- Others (Tip: When in YouTube, click the “360 Videos” button and search for a complete immersive experience)
