# Supplementary material for: Examining the Efficacy of Extended Reality–Enhanced Behavioral Activation for Adults With Major Depressive Disorder: Randomized Controlled Trial
Source: JMIR Ment Health. 2024 Apr 15;11:e52326. doi: 10.2196/52326 (PMC11058556; doi:10.2196/52326)
Supplement: Multimedia Appendix 5 [file mental_v11i1e52326_app5.docx]

**Post-XR Questionnaire**

Please complete this questionnaire once per week.

Date**:**

*Presence*

1. **To what extent did you feel like you were actually inside the virtual experience?**

Not at all            Slightly Moderately           Strongly Very Strongly

1. **To what extent did you feel surrounded by the virtual world you saw?**

Not at all            Slightly Moderately           Strongly Very Strongly

1. **How much did it feel as if you visited another place?**

Not at all            Slightly Moderately           Strongly Very Strongly

Please fill in the below questionnaire. Circle the description that best describes the severity of the specified symptom compared to **your baseline.** For example, if you are normally slightly fatigued, and this experience made you no more fatigued than usual, you would answer *no more than usual*. If this experience made you moderately more fatigued that normal, than you would answer *moderately more than usual.*

| **Nausea** | No more  than usual | Slightly more than usual | Moderately more than usual | Severely more than usual |
| --- | --- | --- | --- | --- |
| **General**  **discomfort** | No more  than usual | Slightly more than usual | Moderately more than usual | Severely more than usual |
| **Stomach awareness** | No more  than usual | Slightly more than usual | Moderately more than usual | Severely more than usual |
| **Sweating** | No more  than usual | Slightly more than usual | Moderately more than usual | Severely more than usual |
| **Increased**  **salivation** | No more  than usual | Slightly more than usual | Moderately more than usual | Severely more than usual |
| **Vertigo** | No more  than usual | Slightly more than usual | Moderately more than usual | Severely more than usual |
| **Burping** | No more  than usual | Slightly more than usual | Moderately more than usual | Severely more than usual |
| **Difficulty concentrating** | No more  than usual | Slightly more than usual | Moderately more than usual | Severely more than usual |
| **Difficulty**  **focusing** | No more  than usual | Slightly more than usual | Moderately more than usual | Severely more than usual |
| **Eyestrain** | No more  than usual | Slightly more than usual | Moderately more than usual | Severely more than usual |
| **Fatigue** | No more  than usual | Slightly more than usual | Moderately more than usual | Severely more than usual |
| **Headache** | No more  than usual | Slightly more than usual | Moderately more than usual | Severely more than usual |
| **Blurred vision** | No more  than usual | Slightly more than usual | Moderately more than usual | Severely more than usual |
| **Dizzy (eyes open)** | No more  than usual | Slightly more than usual | Moderately more than usual | Severely more than usual |
| **Dizzy (eyes closed)** | No more  than usual | Slightly more than usual | Moderately more than usual | Severely more than usual |
| **Fullness of head** | No more  than usual | Slightly more than usual | Moderately more than usual | Severely more than usual |

**Technology Acceptance Model (TAM) of XR Headset:**

*Perceived Usefulness*

1. **Using the VR system would encourage me to do things I wouldn’t normally do**

Strongly Disagree Disagree Neutral Agree Strongly Agree

1. **Using the VR system would give me something to look forward to during the day**

Strongly Disagree Disagree Neutral Agree Strongly Agree

1. **I feel the VR system is useful**

Strongly Disagree Disagree Neutral Agree Strongly Agree

*Perceived Ease of Use*

1. **I feel the VR system is easy to use**

Strongly Disagree Disagree Neutral Agree Strongly Agree

1. **Learning to use the VR system would be easy for me**

Strongly Disagree Disagree Neutral Agree Strongly Agree

1. **My interaction with the VR system would be clear and understandable**

Strongly Disagree Disagree Neutral Agree Strongly Agree

*Attitudes Toward Use*

1. **I like the idea of using this VR system to engage in enjoyable activities**

Strongly Disagree Disagree Neutral Agree Strongly Agree

1. **I have a generally favorable attitude toward using this VR system**

Strongly Disagree Disagree Neutral Agree Strongly Agree

1. **I believe it is a good idea to use this system as part of my treatment process**

Strongly Disagree Disagree Neutral Agree Strongly Agree

1. **I am satisfied with the VR system**

Strongly Disagree Disagree Neutral Agree Strongly Agree

*Intention to Use Technology*

1. **If it were made available to me, I intend to use the VR system**

Strongly Disagree Disagree Neutral Agree Strongly Agree

1. **If it were made available to me, I would continue to use the VR system after completion of this study**

Strongly Disagree Disagree Neutral Agree Strongly Agree

1. **I would adopt the VR system in the future**

Strongly Disagree Disagree Neutral Agree Strongly Agree
